# Investigating the inflammatory response to exposure of ultrafine TiO_2_ particulate matter to HUVECs

**DOI:** 10.1016/j.bbrep.2025.102426

**Published:** 2026-01-06

**Authors:** Laura A.E. Brunmaier, Travis W. Walker

**Affiliations:** Karen M. Swindler Chemical and Biological Engineering Department, South Dakota School of Mines & Technology, Rapid City, SD, USA

**Keywords:** Particle transport, Inflammation, In vitro screening, Particle exposure

## Abstract

Epidemiological studies have indicated that strong causal evidence exists to link the inhalation of particulate matter to the exacerbation of pathology in the cardiovascular system, ranging from myocardial infarction and atherosclerosis to direct cytotoxicity and inflammation. Ultrafine particles are ubiquitous in ambient air, in industrial sites, and in air pollution. When particles are inhaled, deposition can occur in the lungs, and the mechanisms of pathology have been well-studied. However, ultrafine particulate matter can translocate from the lungs into the bloodstream to circulate throughout the body (Choi et al. 2010).Contradictory evidence exists of inflammation and cytotoxicity that is caused from nanoparticle exposure to the endothelium.

When endothelial cells (ECs) are adversely stimulated, they have been shown to secrete cytokines that mediate an inflammatory response. Currently, studies that quantitatively evaluated the secretion of pro-inflammatory cytokines from ECs upon nanoparticle exposure are not accounting for the aggregation that can occur between particles over time and, therefore, are likely exposing cells to a wider range of aggregated sizes. This study evaluates the inflammatory response from ECs after particle exposure, with acute attention devoted to controlling particle aggregation. Specifically, we introduce a protocol that exposes ECs to the particles in a transwell system, where we take advantage of the effects of gravitational settling to expose the ECs only to the smallest fraction of the particles that are in suspension. After 72 h in the transwell assay, we found that the inflammatory response between varying concentrations of particles mirrored the inflammatory response of the positive control of lipopolysaccharide (LPS). These results indicate that the inflammatory response may have a stronger relationship to the particle size than to the concentration of the particles in mass per volume.

## Highlights

• IL-6 secretion may indicate an inflammatory response in HUVECs after TiO_2_ exposure.
• Transwell systems offer a platform to expose cells to the smallest particles in a solution.
• Aggregation alters nanoparticle–cell interactions in vitro.
• TiO_2_ exposure mimics the intensity of LPS-induced cytokine secretion in HUVECs.

## 1 Introduction

Strong causal evidence exists to link the inhalation of particulate matter (PM) to the exacerbation of pathology in the cardiovascular system, ranging from myocardial infarction and atherosclerosis to direct cytotoxicity and inflammation [1], [2], [3], [4]. Studies have shown that inhalation of PM causes airway irritation and inflammation [5], [6], [7], while the relationship between exposure and disease has been found to be largely epidemiological in nature [8], [9]. To investigate the molecular mechanisms and cellular response, various in vivo experiments have been conducted, demonstrating changes in blood pressure, increased rates of atherosclerosis, and occurrence of vascular dysfunction [10], [11].

PM is a sweeping term that classifies matter based on the aerodynamic diameters of the particles that are most commonly suspended in the air that we breathe, while disregarding the composition of the particles. Ultrafine PM (PM0.1) refers to particles with an aerodynamic diameter of 100 nm or less, and they are suspected to have a significant biological effect that is attributed to their relatively high ratio of surface area per mass when compared to colloidal particles [12]. PM0.1 remain suspended in the air for longer periods of time, as the terminal velocity of a particle scales with the square root of its size (e.g., diameter of a sphere), increasing its probability of being convectively dispersed. Further, since the ratio of convective forces to diffusive forces (i.e., the Péclet number) scales with the size of the particle to the fourth power, thermal diffusive motion will typically dominate for these smallest particles, allowing the particles to be infinitely suspended. Once inhaled into the lungs, the particles can travel further into the lungs, eventually depositing into alveoli where the particles can translocate from the lung tissue to the blood stream [13]. Nanoscale particles (NPs) that are inhaled from the ambient air, specifically with a hydrodynamic diameter of 5 nm to 23 nm, as shown by Choi et al. [14] using gel filtration chromatography, resulted in translocation from the lung tissue to the bloodstream, allowing the particles to reach various tissues throughout the body and to potentially cause inflammation. Contradictory to PM0.1 is larger particles. Large particles are more likely to deposit in the nasopharyngeal membranes or in airways, and they can be removed from the tissues via macrophages [15].

Investigating the biological effects of NPs is particularly challenging because the thermodynamic nature of NPs is to form aggregates. The particles in a gas or in a liquid suspension experience Brownian motion, driven by thermal diffusion, which can lead to collisions between particles, promoting aggregation. The number of collisions that are experienced by a particle is also dependent on the concentration or number of nearby particles. Breakup of these aggregates requires a relatively large input of energy to allow for dispersion of a homogeneous distribution of individual particles [16]. Fortunately, aggregation of the NPs decreases the likelihood of NPs translocating to other tissues and increases the likelihood of opsonization, which facilitates clearance [16]. Thus, several parameters should be considered to accurately investigate the biological responses of NPs when they are used in vitro, which include aggregation, particle concentration, the ratio of sedimentation time versus cell-exposure time, and cell-exposure concentration. Each of these contributions are likely to cause discrepancies that are seen throughout the literature, claiming either biological inertness or pathological propensity.

Titanium dioxide (  ) is commonly used in a particle form throughout the cosmetic, paint, and paper industries, and it has been claimed to be biologically inert, having no cytotoxic effects [17], [18], [19]. Suzuki et al. [19] used the MTS [3-(4,5-dimethylthiazol-2-yl)-5-(3-carboxymethoxyphenyl)-2-(4-sulfophenyl)-2H-tetrazolium] assay to measure viability of ECs after 24 h of exposure to concentrations of 1 µg mL^-1^ to 100 µg mL^-1^ of  with no depreciable effects. They observed no measurable increase in expression of monocyte chemoattractant protein-1 (MCP-1) after 16 h of exposure to 1 µg mL^-1^, 5 µg mL^-1^ and 10 µg mL^-1^ of  , indicating that the exposure of  was not causing an inflammatory response in ECs. However, other studies have reported that  inhibits proliferation and induces apoptosis, while inducing expression of adhesion and inflammatory molecules in human umbilical vein endothelial cells (HUVECs) [20].

The data regarding the possible pathological effects or biological inertness of nano- or micro-scale  particles are contradictory. This contradiction may result from the variations that exist in administrative concentrations and routes, as well as a misrepresentation of the particle sizes that are present during experimentation. Studies that carefully evaluate and control particle aggregation, particle concentration, sedimentation time, and the reciprocating exposure concentration on ECs in vitro have yet to be investigated.

Here, we show HUVECs, a common EC line that can be used in vitro, undergoing an inflammatory response that is measured by elevated secretion levels of interleukin-6 (IL-6) after exposure to  particles in a two-dimensional cell culture. IL-6 expression by HUVECs has been previously documented, and the literature supports that an increase in levels of IL-6 expression indicate an inflammatory response [21].

The concentrations of particles that were used in these experiments was guided by information from the Occupational Safety and Health Administration (OSHA) and the National Institute of Occupational Safety and Health, two organizations that are responsible for defining safety guidelines and acceptable exposure limits within the workplace. Interestingly, the acceptable exposure limit of  varies between these two organizations. OSHA defined a threshold of 15 mg m^-3^; however, particles diameters are not defined in this regulation despite the significant variation of biological responses that are dictated by the diameter [22]. NIOSH does not define a threshold, rather classifying  as a possible carcinogen [23]. Chemical companies provide safety data sheets (SDS) which include safe handling guidelines and hazards. For instance, the SDS for  nanopowder from SigmaAldrich states that no data is available for exposure of the particles [24]. On the contrary, the European Chemicals Agency published guidance on the classification and labeling of  stipulating that powder forms or mixtures containing greater than 1% of particles of diameters less than 10 µm must be classified as a category 2 carcinogen by inhalation [25]. This regulatory framework highlights the hazard of ultra fine PM attributed to the potential cell-particle interaction and necessitates studies with particle-size controls to accurately isolate and investigate size-specific effects rather than only mass concentration.

For this investigation, both positive and negative controls were established in each experiment. Lipopolysaccharide (LPS), a bacterial toxin from gram-negative bacteria and a known inflammatory agent to mammalian cells [26], was used as a positive control, giving a comparison to the extent of the inflammatory response that was induced by the  when measuring the secretion of inflammatory cytokines. A negative control of pure growth medium was also included.

Our goal was to isolate exposure to the smallest particle size, specifically in the range of NPs, which we achieved by conducting a two-dimensional experiment of culturing HUVECs in a transwell assay. The transwell membrane insert allowed us to culture cells on top of the membrane with controlled porosity and to submerge it into a bath of cell-culture medium with suspended  particles. The particles with a sufficiently small diameter remained in suspension longer because the dominance of the gravitational force was reduced. EC exposure was limited to only the smallest particles in suspension, while larger aggregates, dominated by gravity, settled from the suspension and therefore did not interact with the cultured ECs.

## 2 Materials and methods

### 2.1 Preparation and characterization of particle suspensions

Nanopowder of rutile titanium(IV) oxide (  ) (Millipore Sigma 637262) with a particle diameter of less than 100 nm, as stated from the manufacturer, was used throughout this study. Exposure of  to light was limited because of its photocatalytic nature. The nanopowder was suspended in deionized water at 1 mg mL^-1^ and autoclaved for 20 min at 121 °C. Particles were then dispersed using a sonicator (Sonics Cibracell VCX750, probe model CV334) for 15 min at an amplitude of 40 %. Suspended particles were taken from the solution and added to cell-culture medium (Lonza EGM Plus, Endothelial Cell Growth Medium Plus BulletKit CC-5035) at concentrations of 5 µg mL^-1^ and 10 µg mL^-1^. Sodium azide  (ThermoFisher, 190380050) at a 0.1% was added to the solutions to prevent bacterial growth.

### 2.2 Particle characterization via dynamic light scattering

The hydrodynamic diameter of the particles was measured using dynamic light scattering (DLS) with a Zetasizer Nano-S (Malvern Instruments) directly after suspending in cell-culture medium or in PBS (phosphate buffered saline).

Two distinct DLS experimental series were conducted. Long-term sedimentation study in PBS and cell-culture medium, 3 trials and 15 scans per trial. The raw values for the volume-weighted size distributions data were exported from the software and plotted using Python. High-resolution time-evolution study in cell-culture medium, 5 trials and 15 scans per trial. The raw values for number-weighted size distributions were exported from the software and plotted using Python.

The first measurements were taken at 0 h, immediately following sonication and suspension preparation. The suspension was kept in the same cuvette, then covered with tin foil to protect the particles from light. The samples were allowed to rest undisturbed before the measurement was taken again to prevent jarring of any sedimented aggregates.

### 2.3 Conditions of cell culture and assays of cell exposure

**Fig. 1:**
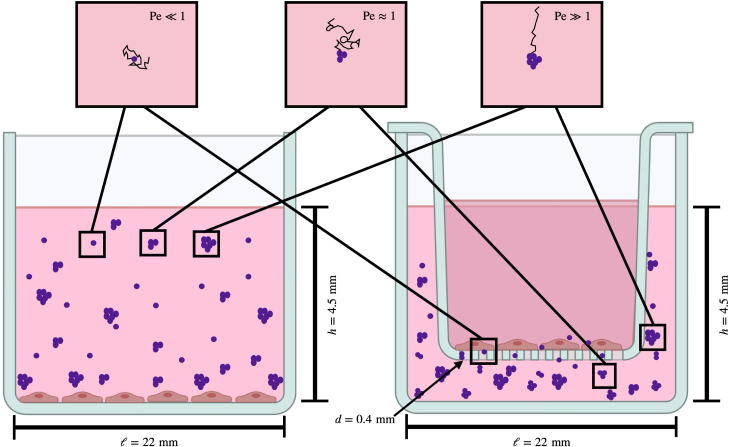
Schematics of exposure assays. HUVECs were cultured in each assay for 24 h; then, the medium was exchanged either with LPS (positive control), with a particle suspension, or with culture medium (negative control). In both assays, particles will form aggregates of various diameters. The size of these aggregates are a large determining factor to whether the aggregate will be more susceptible to convective or diffusive forces. The cell supernatants were collected, and cytokine secretion was measured via ELISA. (Left) General assay of cells that are cultured at the bottom of a cell-culture well where particles are suspended in the medium above the cells. The cells are able to interact with all sizes of particles in the solution. (Right) Advanced assay of cells that are cultured on the top of the porous membrane in a transwell, and the insert was submerged into a bath of the particle-medium suspension. Some particles will be able to overcome gravitational settling and membrane filtering (d≤400nm) to translocate through the membrane and interact with the cells. Images created in BioRender.com.

HUVECs from pooled donors (Lonza, C2519 A) were cultured in Lonza EGM-Plus Endothelial Cell Growth Medium-Plus Bulletkit (Lonza CC-5035) at 37 °C and 5 mol % of  . Cells were passaged with trypsin-EDTA (2.5 g L^-1^; Sigma-Aldrich T4049), 1× PBS, and trypsin neutralizing solution (Lonza CC-5002). Cells for experimentation were used at passage 3 with a minimum of two recovery passages from cryopreservation. At passage 3, the cells were seeded at 2 × 10^4^ cells cm^-3^ into well plates for tissue culture, as shown in Fig. 1 (Left), or in a transwell membrane with a polyester membrane (Corning 3460; pore diameter of 0.4 µm), as depicted in Fig. 1 (Right). The cells were cultured for 24 h before experimentation began to allow for cells to attach to the culturing substrate. The initial medium was replaced with the respective particle-laden medium that was composed of 5 µg mL^-1^ and 10 µg mL^-1^ of  , 1 µg mL^-1^ of LPS as a positive control, and culturing medium as a negative control. The medium supernatant was collected at 0 h, 24 h, 48 h and 72 h.

### 2.4 ELISA and data analysis

Supernatants of cell medium were collected from both the well plates and the transwell plate at 0 h, 24 h, 48 h and 72 h. Levels of four cytokines that are known to be related to inflammation were measured – vascular endothelial growth factor (VEGF) [27], interleukin-6 (IL-6) [28], interleukin-8 (IL-8) [29], and monocyte chemotactic protein-1 (MCP-1) [30] – using an enzyme-linked immunoassay or ELISA (Biogems, Westlake Village, CA).

### 2.5 Scanning electron microscopy/ energy dispersive X-ray spectroscopy transwell sample preparation and imaging

After completing the exposure time point, the transwell insert was removed from the culture plate and fixed with 4% paraformaldehyde (Sigma-Aldrich J61899.AP) for 10 min. The samples were rinsed 3 times with PBS and allowed to air dry. After drying, the membrane was cut out with a razor blade and mounted on an SEM post. The samples were sputter coated under high-vacuum for 10 nm carbon film deposition with a Leica ACE600 to aid in surface charging. SEM was conducted with a Thermo-Scientific Helio 5 CX at 20 kV accelerating voltage, 1.4 nA current, using an Everhart–Thornley detector (ETD). The EDS was an Oxford Ultim Max100 running Oxford Aztex version 6.1 at 0.6 nm resolution.

## 3 Results

### 3.1 Characterization of nanoparticle suspensions

Given the relatively small length scale of NPs, certain “aspects of smallness” [31], such as the presence of mobility due to thermal kinetic energy and an absence of inertial effects, become prevalent. Since body forces (e.g., gravitational forces) scale like the cube of the length scale of the object while colloidal surface forces (e.g., drag forces) scale like the square of the length scale, gravitational effects become increasingly negligible as the size of the particle is decreased. Thus, thermal transport dominates over convective transport of NPs in a system, as explored further in Section 3.2, which should cause the particles to remain suspended longer, randomly diffusing and sampling the space.

During this diffusive process, particles may collide. Considering the increased dominance of surface forces such as electrostatic interactions and relative surface energy, the collisions can lead to aggregation, which results in a lower surface energy yet strong cohesion forces [32]. NP suspensions are commonly sonicated to disperse NPs into the solution; however, complete breakup of aggregates that form when adding a particle powder to a solution is particularly challenging because of these cohesion forces that are experienced between particles. Additionally, collisions between particles within the solution can occur during the entire duration of the experiment, which may result in continuous aggregation and sedimentation. Therefore, DLS measurements are taken just preceding cell exposure to determine the particle-size distribution of the suspension.

Another important consideration is that the medium in which the particles are suspended for this experiment is a complex solution, containing proteins, sugars, growth factors, salts, and amino acids, among other components that are used for the culturing of cells. The interactions that occur among these components and the particles that are dispersed in suspension are not well-defined. A possible interaction that we suspect may occur is protein adsorption to the surface of the particles, which may either deter particle aggregation or enhance it, depending on the protein–protein and protein–particle interactions. Thus, we incorporated PBS as a control for the DLS measurements because of its relative simplistic composition.

Typically, an initial DLS measurement of the particle suspension is taken without consideration of the dynamic particle interactions that occur in the solution, which can effect the size distribution over time [19], [33]. After collecting the data immediately after suspending the particles (t=0h), the suspensions were left undisturbed for t=72h to replicate the conditions of cell exposure. These timepoints also correlate with the beginning and the end of the experiments of cell exposure (Fig. 2).

At t=0h, average aggregate diameters ranged from 500 nm to 700 nm, which is significantly larger than the average diameter of d=100nm that was reported by the manufacturer. When DLS measurements were repeated, we observed evidence that significant sedimentation with probable aggregation occurred in both PBS and medium. The distribution for the PBS suspension had shifted to an average aggregate diameter of around d=250nm, and the distribution for the medium suspension shifted below d=100nm. The difference in hydrodynamic diameter between the two suspensions suggests that protein–particle interactions occurred in the medium, which likely altered the aggregation behavior over time. While proteins in the culture medium can form a corona that stabilizes NPs, complex adsorption dynamics may also influence apparent size distributions and sedimentation patterns.Fig. 2DLS data at t=0h and t=72h for particles that were suspended in (a) medium and (b) PBS at an initial concentrations of 10 µg mL^-1^. The blue region indicates the range of the particle diameters as indicated by the manufacturer. The red region indicates the anticipated particle size where gravitational forces dominate and sedimentation occurs. After 72 h, the hydrodynamic diameter of the particles that remained in suspension were measured again. In the medium suspension, the average diameter of the particles was d<100nm; however, the amount of particles remaining in suspension was less than the amount of particles that were initially dispersed at t=0h because of sedimentation. The PBS-particle suspension reported an average diameter significantly larger than the medium suspension. The distributions are averages of n=3 trials.Fig. 2

**(a):**
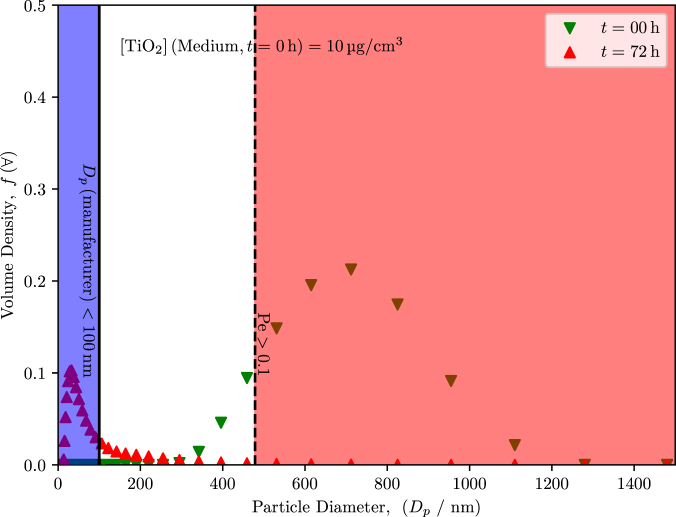
Medium.

**(b):**
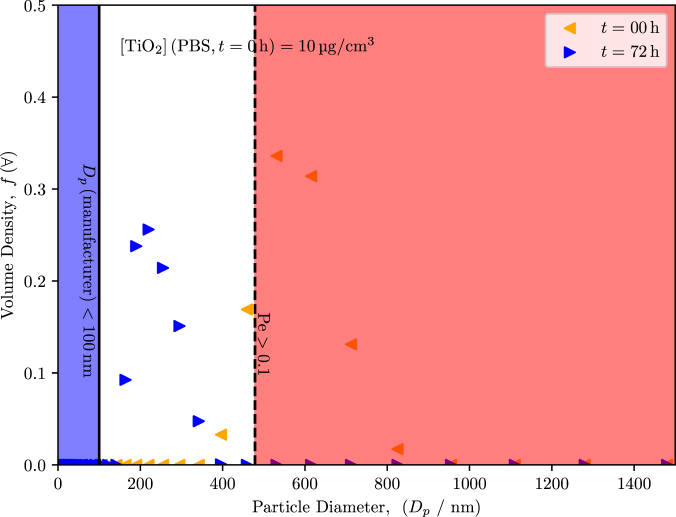
PBS.

### 3.2 Microhydrodynamics

To define a mechanism of transport that occurred in our suspensions of dispersed particles in cell medium, we considered the particle-size distribution within our suspension, the particle sizes that are dominated by gravitational or thermal forces, and the particle-settling velocity that results from the span of the measured hydrodynamic diameter.

**Gravitational Péclet number.** The Péclet number (Pe) is a crucial dimensionless number to consider in this study, as it describes the dominant transport of NPs to the cells in a system. The Péclet number quantifies the ratio between convective transport and diffusive transport, such that

(3.1) $$\mathrm{Pe}=\frac{4\pi \Delta \rho ga^{4}}{3k_{B}T},$$

for this gravitational-driven system, where Δρ is the difference between the particle density and the fluid density, g is the magnitude of the acceleration of gravity, a is the particle radius, $k_{B}$ is the Boltzmann constant, and T is the absolute temperature. During sedimentation, the gravitation force is the driving force of convection, while a critical Péclet number (${\mathrm{Pe}}_{c}$) exists at unity, implying a balance between competing forces. A Péclet number that is greater than 1 indicates that convective transport is predominating in the system and that particles of this radius and larger have a tendency to sediment directly. A Péclet number that is smaller than 1 indicates that diffusive transport is predominating and that particles of this radius and smaller should remain in solution longer, diffusing about randomly. Representative particle paths at different Péclet numbers are depicted in Fig. 1.

Thus, for a specific system, a critical radius (or corresponding diameter) can be calculated from Eq. (3.1) by substituting the critical Péclet number and by rearranging to solve for the particle radius. Given our experimental conditions, we calculated that the critical Péclet number of 1 corresponded with a critical diameter of 850 µm. In practice, a Péclet number of 0.1 or greater will lead to substantial sedimentation, corresponding with a critical diameter of 480 nm, which was indicated on Fig. 2. Relating the DLS data to Eq. (3.1), at t=0h, the average diameter was 500 nm to 700 nm, meaning that a large fraction of the particles in suspension have the potential to undergo sedimentation from convective forces. However, at t=72h, the average diameter was less than d=100nm, meaning that the remaining particles in solution experience predominantly thermal forces, which leads to the random sampling of the container volume via Brownian motion.

**Terminal settling velocity.** Our DLS results (Fig. 2) indicated that sedimentation and probable aggregation does readily occur; therefore, the Péclet number provides the necessary assessment of transport in this system. Since some particles would assuredly sediment from the suspension during the duration of our assay, determining the rate at which settling would occur via Stokes law for drag on a sphere can provide context for our exposure experiments. The terminal settling velocity (v) under creeping flow can be determined for a given particle diameter, such that

(3.2) $$v=\frac{2a^{2}\Delta \rho g}{9\mu },$$

where μ is the viscosity of the fluid [34], assumed to be approximately 0.69 mPa⋅s for the aqueous media at 37 °C.

If we consider the critical diameter of 850 nm, the settling velocity of a freely-suspended particle in the system is 1.8 × 10^-6^ m s^-1^, which would imply that a particle that is in a cuvette on the order of 1 cm would completely settle in about 92 min without Brownian motion. However, this settling time would decrease with larger diameters and for particles that are initially located closer to the bottom of the container. We can apply this calculation to our experimental conditions, where the cells are cultured and adhered to the bottom surface of a well with a diameter of 22 mm and with a fluid height of 4.5 mm. Particles of the critical diameter would take about 41 min for gravity to drive the particles at the top of the domain to the cells that are cultured at the bottom of the well, assuming that collisions with other suspended particles did not significantly drive the descending particle from a downward linear path and that the Brownian motion was negligible.

**Fig. 3:**
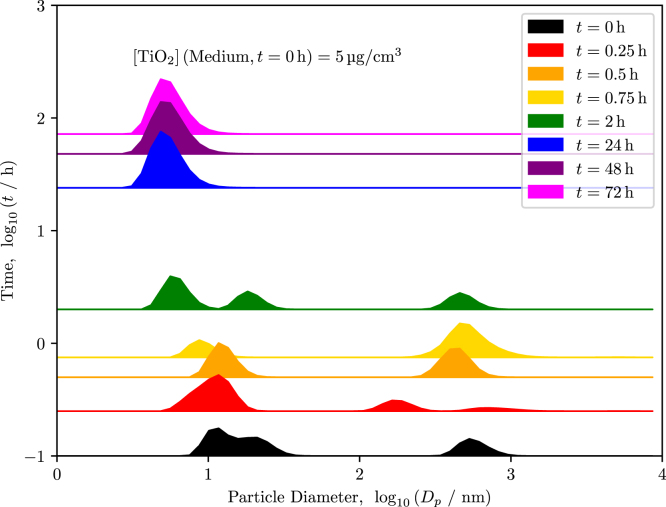
DLS data at t∈{0,0.25,0.5,0.75,2,24,48,72},h for particles that were suspended in medium at an initial concentration of 5 µg cm^-3^. The logarithm of the particle diameters is plotted on the abscissa with the logarithm of the time plotted on the ordinate. The maximum height of the distributions is normalized to unity on the ordinate, and the range of the distributions is exemplified for the data at t=72h, where the maximum is shown as a dotted, magenta line. After 24 h, the hydrodynamic diameter of the particles appeared to be on the order of 10 nm.

DLS measurements performed over 72 h confirm rapid sedimentation of the larger fraction of the  suspension, Fig. 3. To clearly resolve the bimodal size distribution and prevent the small-particle population from being masked by the scattering intensity of larger aggregates, data are presented in number-weighted form on a log–log scale (particle diameter $D_{p}$ and time t). At t=0, the suspension exhibits a pronounced bimodal distribution with a dominant mode centered at 500 nm to 700 nm (well above the Pe≈0.1 threshold of 480 nm) and a smaller mode below 100 nm. Within the first 2 h, the large-particle mode almost completely disappears from suspension, consistent with strong gravitational settling of aggregates as described by Eq. (3.2). By 24 h, only the small-particle population remains detectable, stabilizing at a hydrodynamic diameter of approximately 10 nm corresponding to a size regime where Brownian diffusion dominates transport. This rapid shift from sedimentation-driven to diffusion-driven behavior is supported by Eqs. (3.1), (3.2).

The calculations of the Péclet number and terminal settling velocity utilize the particle density as stated from the manufacturer. Other computational methods one may consider is the in vitro sedimentation, diffusion and dosimetry model (ISDD) [35]. However, this material value is not the same density of an aggregation of particles, as they tend to exhibit a porous nature. Therefore, this density difference is a source for error that should also be considered.

**Particle Transport in Biological Assays.** Many of the assays that are used to determine the effects of NPs on cells exposure are conducted by culturing cells in a two-dimensional (2D) well, where the standard culturing medium is exchanged with a medium that contains the suspended particles. However, sedimentation of NP aggregates driven by gravitational convection dramatically increases particle–cell contact and uptake at the well bottom. This phenomena is governed by the terminal settling velocity Eq. (3.2). Yet, if the goal of the research is to expose cells to measure a response that is caused by the smallest particles in the suspension, then the 2D configuration of exposure means that the smallest particles are the least likely to interact with the cells as discussed in Section 3.2. Therefore, any resulting data aiming to characterize a cell response are less indicative of the small, biologically translocatable particles and more indicative of the larger aggregates that sediment to the bottom of the well and directly contact the cells. The lack of control of particle size in 2D assays may be a strong contributing factor to challenges behind in vitro reproducibility and contradicting data.

### Inflammatory response of HUVECs in tissue-culture wells with 

3.3

Secretion of inflammatory cytokines correlates to the onset of the inflammatory response [21]. Numerous previous studies have reported the particle–cell interaction and response. Montiel et al. [20] investigated particle exposure to HUVECs and found a decrease in the proliferation and an increase in apoptosis after particle exposure. They also saw an increase in expression of adhesion molecules, which can indicate the beginning of an inflammatory response. Han et al. [33] found an inflammatory response that was caused by exposure of  NPs that stemmed from an increase in mRNA levels of vascular cell adhesion protein-1 (VCAM) and monocyte chemoattractant protein-1 (MCP-1), which they attributed to oxidative stress. Importantly, particles with a diameter of 5 nm, as reported by the manufacturer, were used in this work, and DLS results from this study indicate that the particles in solution formed aggregates ranging from 250 nm to 396 nm.

The HUVECs in these experiments were screened for the secretion of IL-6, IL-8, and MCP-1 after being exposed to culture media with suspended  particles or culture medium with LPS functioning as the positive control for its known initiation of an inflammatory response [26]. We were also interested in levels of vascular endothelial growth factor (VEGF) because of the importance of increased vascularization during inflammation [36]. The purpose of the design of this experiment was to screen for which inflammatory cytokines that are both detectable in vitro and specific to HUVECs. To determine if a positive inflammatory response was exhibited during the experiment, the secretion level of the cytokine was compared to both the positive and negative control.

Microscope images of the cells, shown in Fig. 4, were taken at 72 h after the respective exposure. From the brightfield images that were taken, significant aggregation of the particles in the cells are shown as the black spots in the image. The collection of aggregates in the cells visually depicts the significant interaction that the cells have with the particles in suspension.

**Fig. 5:**
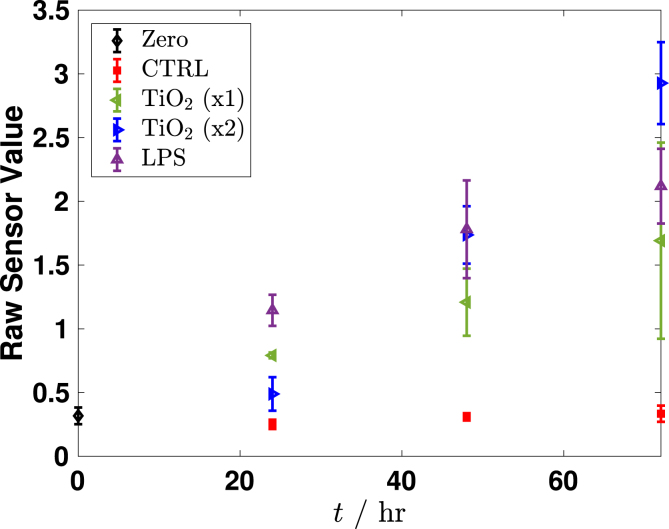
Screening results of IL-6 inflammatory cytokine secretion from HUVECs from 0 h to 72 h cultured on a tissue-culture well plate. The ELISA output is plotted as raw sensor value to illustrate relative signal intensity as a means to screen for cytokine secretion qualitatively. HUVECs were exposed to [LPS]=1μg/mL as a positive control or to two different particle suspensions, where  (x1) refers to  and  (x2) refers to  . The negative control was only cell-culture medium. All exposures demonstrate an elevated IL-6 secretion when compared to the negative control. Particle exposure at 72 h was comparable to the positive control. Each data point represents n=3 replicates with associated error bars representing standard deviation.

Supernatants were collected from the wells at 0 h to establish a pre-exposure baseline for cytokine levels. Sample collection was continued at time points 24 h, 48 h and 72 h. We found that no appreciable increases existed in the levels of IL-8, MCP-1, or VEGF when compared to the positive and negative controls. However, levels of IL-6 showed a marked increase that was comparable to the positive control, especially at 48 h and 72 h. Fig. 5 shows the results of the ELISA measurements of IL-6. The ELISA results from this initial screening experiment are reported as raw sensor values to qualitatively compare cytokine signal intensity among conditions. These values confirm the strong IL-6 response relative to other cytokines and were used to guide the subsequent quantitative transwell experiments.Fig. 4Brightfield images of HUVECs that were taken 72 h after exposure to (a) negative control, (b) , (c) , and (d)[LPS]=1μg/mL. Dark regions correspond to  particle aggregates interacting with cells captured in (b) and (c). These images are provided as qualitative illustrations of particle–cell interactions rather than quantitative assessment of viability or proliferation. Apparent differences in cell confluency appears significantly different between the cells that were exposed to particles or to LPS when comparing to the negative control cells. These results may indicate other deleterious effects that are caused by exposure.Fig. 4

**(a):**
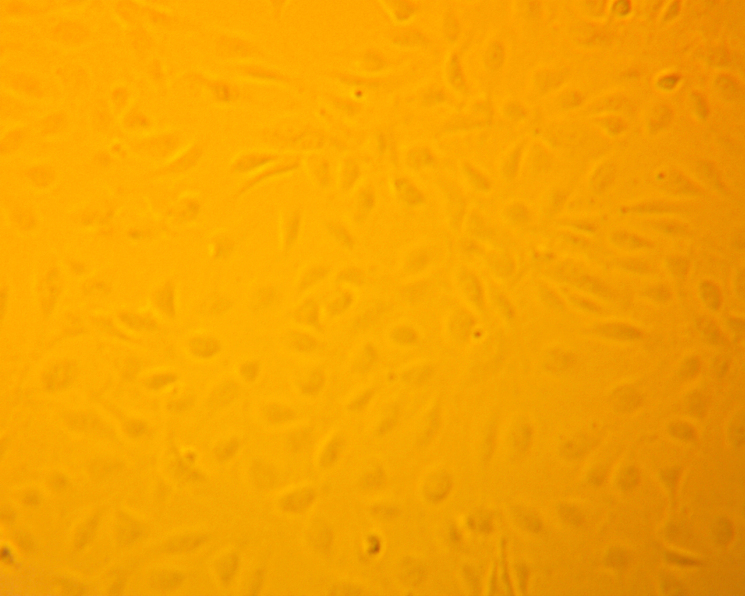
Negative control.

**(b):**
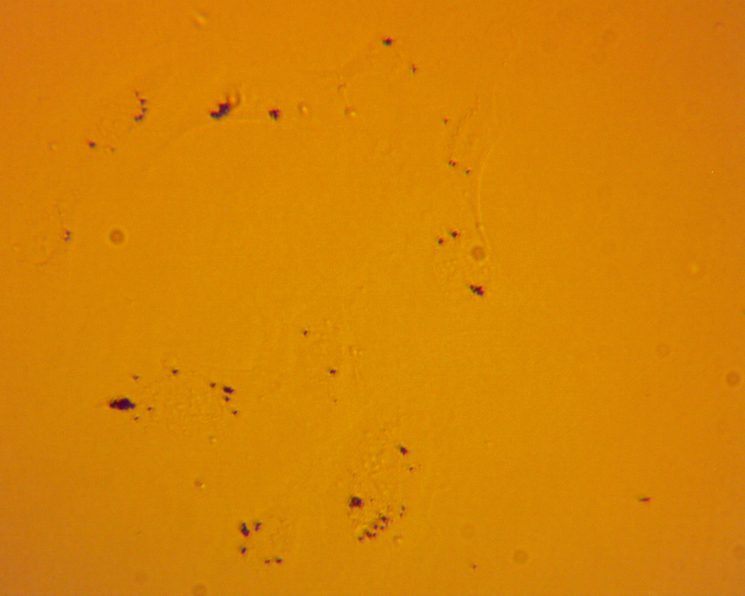
.

**(c):**
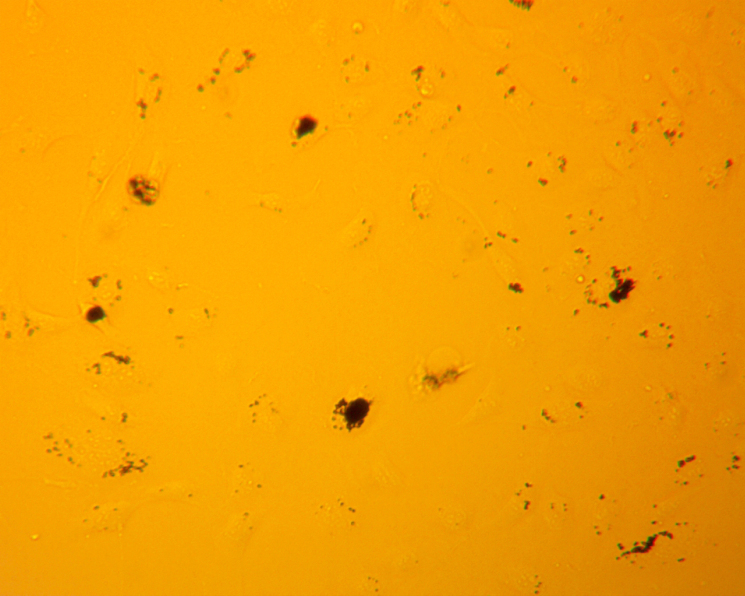
.

**(d):**
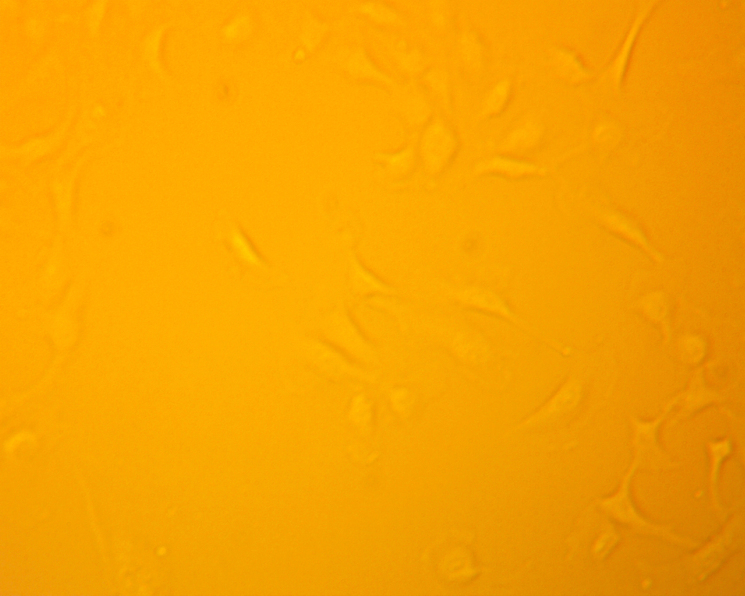
[LPS]=1μg/mL.

Cytokines often exhibit pleiotropic tendencies, but IL-6 has been shown to be characteristically inflammatory in HUVECs [37]. Huang et al. [21] quantitatively showed an increase in IL-6 expression by HUVECs that were exposed to Dengue virus (DV) and in sera of DV-infected patients, suggesting a possible link to IL-6 levels and the vessel permeability that occurs during hemorrhaging. Furthermore, two other studies indicate that IL-6 expression has been evidenced in unstable angina and myocardial infarction [38], [39].

Our observation of no significant increase in levels of MCP-1 in HUVECs after exposure of  particles (data not shown) aligns with prior work by Suzuki et al. [19]. However, contradictions remain, where other studies that were completed with porcine pulmonary artery cells [33] and in HUVECs [40] do indicate an increase in expression of MCP-1. The discrepancy contributing to these results is not clear, but it may be attributed to differences in concentration, particle composition, particle size, or particle shape, as well as the cell species that were used during the study. Concentration can vary from 1 µg mL^-1^ to 200 µg mL^-1^ between different studies, which have a weak correlation to the environmental exposures discussed here [19], [33], [40]. Liu et al. [40] indicated elevated levels of IL-8 and MCP-1 in their control samples and no significant increases in either MCP-1, IL-8, or IL-6 until they increased the exposure to 200 µg mL^-1^.

Contradictions between studies are abundant. The cause for these contradictions may be attributed to multiple variables, including particle composition, size, shape, concentration, cell type, and exposure duration. Our data indicated a clear increase in signal from IL-6 as a result of exposure to  at both concentrations and to LPS. Therefore, we moved forward with our next exposure system, where we intend to quantify the intensity of the responses of IL-6 from exposure to NPs of  and to LPS.

### 3.4 Controlling exposure to sufficiently small particles in a transwell system

NPs with diameters of d≤100nm have been hypothesized to have a higher propensity to cause adverse biological effects than larger diameter particles [41]. However, testing for these effects is particularly challenging in vitro because particles in this size range readily aggregate into larger aggregates. These aggregates have a larger diameter, growing to sizes that results in increased sedimentation from the fluid medium. Consequently, cells that are cultured in vitro under static conditions have higher exposure to the cluster of particles because the clusters have a higher rate of settling than the particles with a smaller diameter. The heterogeneity of particle diameters and fluid height in prepared in vitro experiments makes the actual level of exposure challenging to quantify.

Our goal was to isolate cells from exposure to larger aggregates and to expose the cells only to particles of smaller diameters that specifically remain suspended indefinitely in the culture medium. A transwell membrane that is commonly used for cell culture allowed us to culture the cells on top of the membrane insert, and the culture medium with  particles or with LPS were added to the well below the membrane, as depicted in Fig. 1. This system allowed the larger aggregates to settle away from the cells to the bottom of the well, limiting interaction with the cultured cells. However, the particles with the smaller diameter, which are less affected by gravity, remain suspended in solution. Only the suspended particles that are small enough to pass through the pores of the membrane could interact with the cells. The supernatants of the cell were collected at the time points of 0 h, 24 h, 48 h and 72 h to investigate the inflammatory cytokine secretion of IL-6 from the cells.

**Fig. 6:**
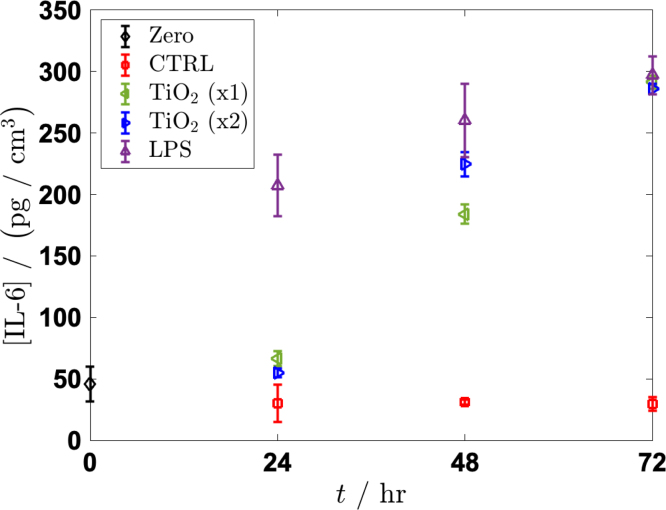
Secretion of inflammatory cytokine IL-6 from 0 h to 72 h in the transwell system. HUVECs were exposed to [LPS]=1μg/mL as a positive control or to two different particle suspensions, where  (x1) refers to  and  (x2) refers to  . The negative control was only cell-culture media. At 24 h, the cells that were exposed to LPS showed a significantly higher response of IL-6 when compared to the cells that were exposed to the particles in suspension. By 48 h, the response of IL-6 to LPS and to both concentrations of [  ] appeared consistent. By 72 h, little difference existed in the levels of IL-6 between the cells that were exposed to the positive control and the cells that were exposed to the particles in suspension. Each data point represents n=3 replicates with associated error bars representing standard deviation.

Our results indicate a clear response of secretion of IL-6 from the HUVECs, as shown in Fig. 6. The response is not significantly different from the negative control until 48 h after exposure. The time delay that was observed can likely be attributed to the time that the suspended particles required to randomly navigate through the porous membrane of the transwell and to eventually interact with the cultured cells. At 48 h, the levels of IL-6 secretion were elevated when comparing the negative control, but by 72 h after exposure, the secretion levels of IL-6 from  and LPS exposure were similar. Importantly, IL-6 secretion level converged across the conditions at the upper detection limit of the ELISA used in this study.

Scanning electron microscopy (SEM) with energy-dispersive X-ray spectroscopy (EDS) confirmed the presence of Ti within the ECs following exposure to  particles Fig. 7. Elemental mapping identified distinct signals of Ti that were localized to regions that are consistent with the intracellular or surface-associated aggregates that were also observed in the brightfield images in Fig. 4. These results verify that  NPs traveled from the basal compartment, through the membrane pores, then were in direct contact with the ECs cultured on the transwell membrane. This evidence supports that the observed IL-6 secretion corresponds to a true particle–cell interaction. Further, the confirmation of Ti supports internalization of  NPs by ECs further supporting that the inflammatory response that was measured through IL-6 secretion.

The detection of Ti within the cell regions by SEM-EDS supports internalization of  NPs because any particles on the top surface, and are not embedded, most would either be removed during rinsing and fixation. Additionally, EDS inherently has a shallow interaction depth, however, sharpness of the particles detected suffered which supports the justification that the Ti is beneath the immediate surface of the cell, and attenuating the particle boundary contrast. These results may elucidate to the powerful effects that the size of particles has rather than only the concentration of particles to which the cells are exposed.

Fig. 7SEM EDS for HUVEC that were grown in the transwell system with Media plus different concentrations of  for t=48h. (Top) Results of SEM. (Bottom) Corresponding results of EDS for only Ti for the same locations as Top. (a) The concentration was  . (b) The concentration was  . Length scales are provided, corresponding to both the top and bottom figure in the same column.Fig. 7

**(a):**
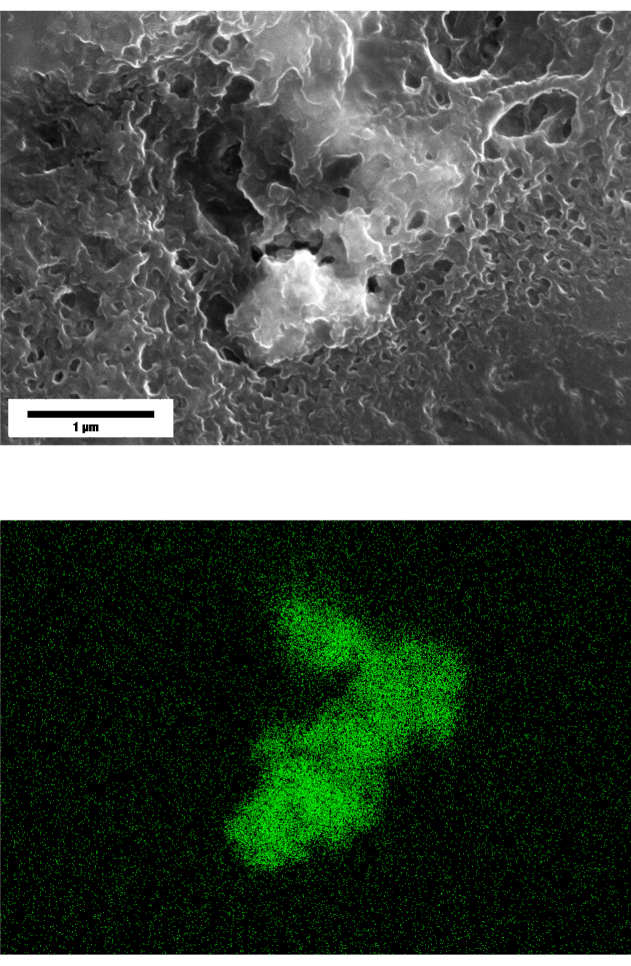
.

**(b):**
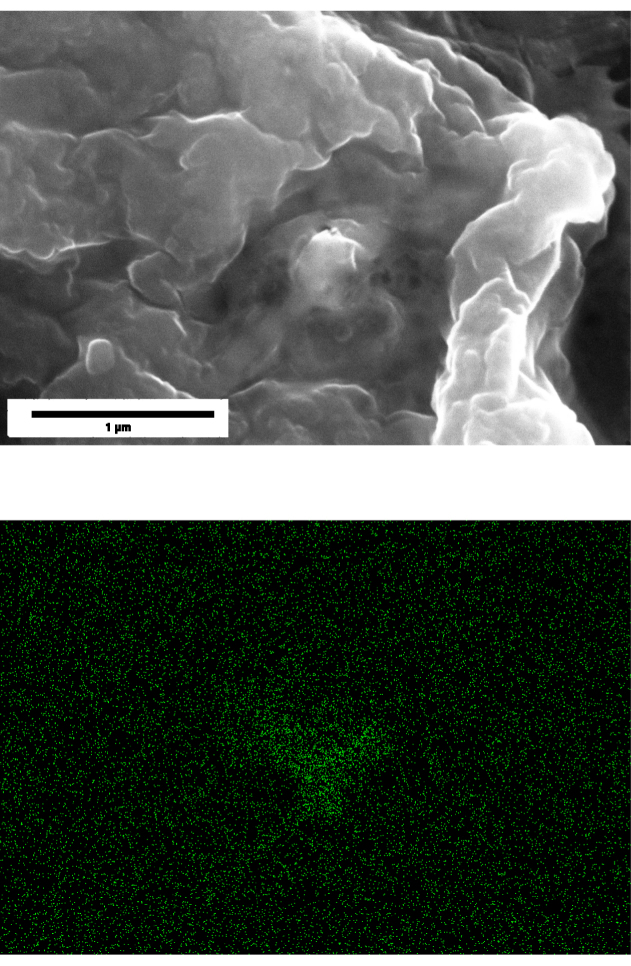
.

## 4 Conclusion and future work

To overcome limitations with simple exposure assays, we designed an experiment utilizing a transwell membrane system to expose HUVECs to particles with the smallest fraction of diameters that were suspended in a solution and measured the levels of secretion of an inflammatory cytokine, IL-6, from the exposed cells. As particle diameters reach the nanoscale, the level of exposure that is capable of producing adverse effects is likely lower, possibly resulting from the high ratio of surface area to volume. However, the exact effects are not well understood in relation to the type, concentration, surface chemistry, etc. Our adaptation of the transwell system allowed us to compare to the traditional method of measuring the effects of NPs on cells, inspiring new routes to exploring in vitro nanoparticle exposure. The absence of MCP-1 and IL-8 induction during the early screening steps combined with the delayed response in the transwell exposure design supports data indicating that  NPs induce an inflammatory response at exceeding low concentrations. Further, this work begins to elucidate the elevated cardiovascular disease that has been reported in epidemiological studies of populations that are living in polluted areas. However, nanoparticle exposure of  is also experienced by workers in the paper, paint, and other essential industries.

Future work will focus on adapting the transwell design into a more physiologically relevant co-culture model incorporating pulmonary epithelial cells adjacent to endothelial cells. Crosstalk between these cell layers following exposure to the smallest NPs remaining in suspension may amplify the inflammatory response, but could also provide a robust platform to investigate nanoparticle deposition and translocation while minimizing confounding effects from larger aggregates [42].

## CRediT authorship contribution statement

**Laura A.E. Brunmaier:** Designed methods with guidance from T.W., Prepared the manuscript text and figures. **Travis W. Walker:** Provided formatting, aided in data analysis and figure preparation, and provided edits.

## Ethics approval and consent to participate

Not applicable.

## Funding

The authors would also like to acknowledge the National Science Foundation Graduate Research Fellowship Program, 2021321384, and NIOSH-funded MCOHS-ERC Pilot Projects Research Training Program (OH008434) for funding this work. The contents of this effort are solely the responsibility of the authors and do not necessarily represent the official view of the National Institute for Occupational Safety and Health, Centers for Disease Control and Prevention, or other associated entities.

## Declaration of competing interest

The authors declare the following financial interests/personal relationships which may be considered as potential competing interests: Laura Brunmaier reports financial support was provided by MCOHS-ERC Pilot Projects Research Training Program. Laura Brunmaier reports financial support was provided by National Science Foundation Graduate Research Fellowship Program. If there are other authors, they declare that they have no known competing financial interests or personal relationships that could have appeared to influence the work reported in this paper.

## Data Availability

Data will be made available on request.
