# Corrigendum to “Investigating the Inflammatory Response to Exposure of Ultrafine TiO_2_ Particulate Matter to HUVECs”*Biochemistry and Biophysics Rep.,* volume 45 (2026) 102426

**DOI:** 10.1016/j.bbrep.2026.102462

**Published:** 2026-01-22

**Authors:** Laura.A.E. Brunmaier, Travis.W. Walker

**Affiliations:** Karen M. Swindler Chemical and Biological Engineering Department, South Dakota School of Mines & Technology, Rapid City, SD, USA

The authors regret to add the following information as acknowledgement of funding.

"Research reported in this publication was supported by the National Institute Of General Medical Sciences of the National Institutes of Health under Award Number P20GM103443 (SD INBRE). The content is solely the responsibility of the authors and does not necessarily represent the official views of the National Institutes of Health.”

The authors would like to apologise for any inconvenience caused.

